# Hemangioma in minor salivary glands: real or illusion

**DOI:** 10.1186/1746-1596-1-21

**Published:** 2006-08-17

**Authors:** Irulandy Ponniah, Palani SureshKumar, Kaliappan Karunakaran, Kolappan A Shankar, Mayelam G Kumaran, Lakshmi Narasimhan Preeti

**Affiliations:** 1 Assistant Professor, Department of Oral & Maxillofacial Pathology, Tamil Nadu Government Dental College & Hospital, Chennai 600 003, India; 2 Assistant Professor, Department of Oral & Maxillofacial surgery, Tamil Nadu Government Dental College & Hospital, Chennai 600 003 India,; 3 Associate Professor, Department of Oral & Maxillofacial Pathology, JKK Natarajah Dental College & Hospital, Nammakal, Tamil Nadu, India; 4 Oral & Maxillofacial Pathologist, In Private Practice, Chennai, India; 5 Postgraduate Student, Department of Oral & Maxillofacial Pathology, Tamil Nadu Government Dental College & Hospital, Chennai 600 003, India

## Abstract

Hemangioma is a common soft tissue lesion in the head and neck region. Hemangioma in the context of minor salivary glands is rarely encountered in surgical pathology practice, and for this reason most pathologist are often unfamiliar with its histomorphological features. We report a rare histological finding of salivary gland structures within a cavernous hemangioma, which may or may not have originated in the minor salivary gland.

## Background

Though hemangioma is a common soft tissue lesion in the oral and maxillofacial region, pathologists are often unfamiliar with the histomorphology of hemangioma in the context of salivary glands [1], especially minor salivary glands. According to the AFIP files, hemangioma is the most common non-epithelial tumor with an incidence of 30% in the major salivary glands [2]. Although arteriovenous malformation of a minor salivary gland have been reported [1], the incidence of hemangioma in minor salivary gland is difficult to ascertain. One of the main reason for the under reporting of hemangioma is the fact that they are rarely biopsied [1]. Even in those biopsied, the diffuse distribution of minor salivary glands and the lack of distinct anatomic boundaries preclude unequivocal evidence of origin in the salivary gland connective tissue stroma [2]. The purpose of the present article is to report a rare histological finding of minor salivary gland elements within a hemangioma and its diagnostic dilemma.

## Case report

This is a report of a 55 year old female who presented with pain over the right side of the face for the past 3 years. She stated that the pain evolved spontaneously and is continuous, often disturbing her from sleep. She also stated that she did not get much relief from routine analgesics. Emotionally she was very much depressed, as she had been evaluated by a number of general and oral physicians. But the diagnosis remained elusive. Her attending dental care provider had extracted few teeth, presumably on the assumption that the pain was of dental origin. However, there was no relief and hence she presented to us.

On extra oral examination no anomalies were detectable. Intra oral examination showed a brownish red discoloration, measuring 1 × 0.75 cm on the lingual aspect of the right retro molar region. The surface of the lesion was slightly elevated and irregular. There was no pain on palpation but it bleed. The lesion blanched on pressure but immediately refilled upon release. Based on the clinical appearance, a vascular lesion was high on the diagnostic considerations. Panoramic radiograph showed no significant finding. The lesion was excised with proper precautions. During excision the bleeding was little more than normal and continued for 15 minutes, but was arrested following pressure pack application. In the next few days following excision the patient reported that the pain had subsided considerably.

**Figure 1:**
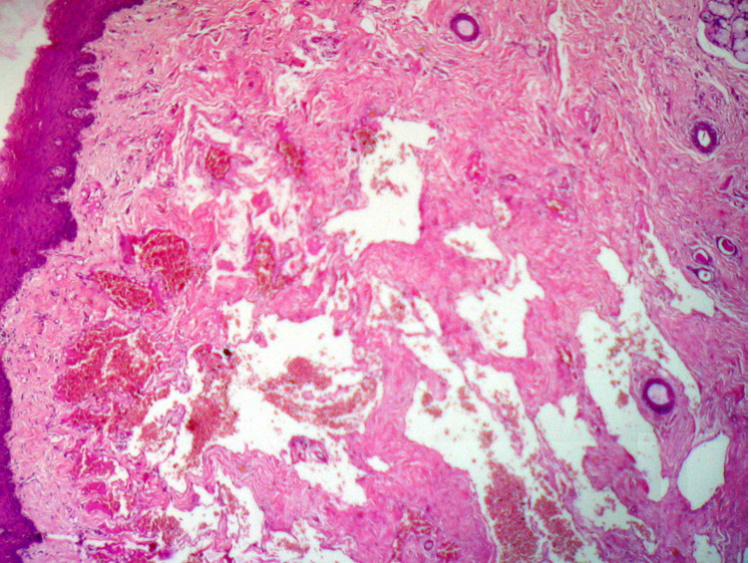
Shows the major part of the lesion with vascular proliferation and salivary gland structures (Original magnification × 24).

**Figure 2:**
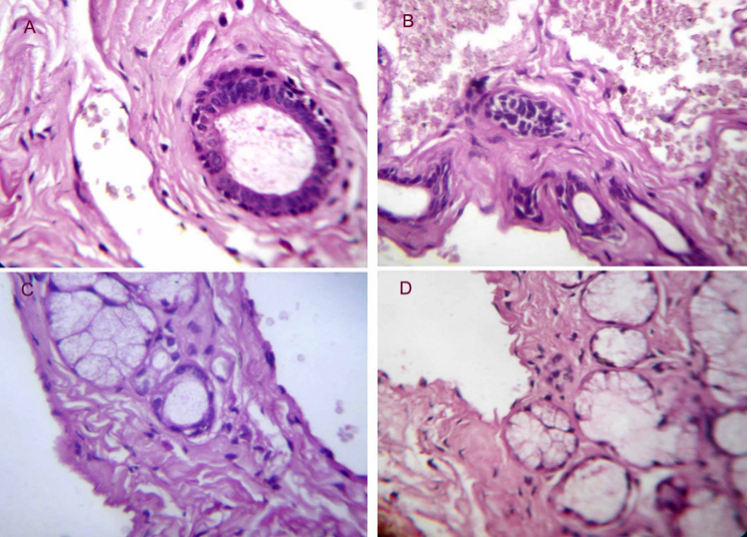
Shows persistence of salivary ducts and dilated, blood filled vessels lined by flattened endothelium in A and B, entrapped mucous acini amid vascular proliferations in C and effaced acinar units in D (Original magnification × 400).

**Figure 3:**
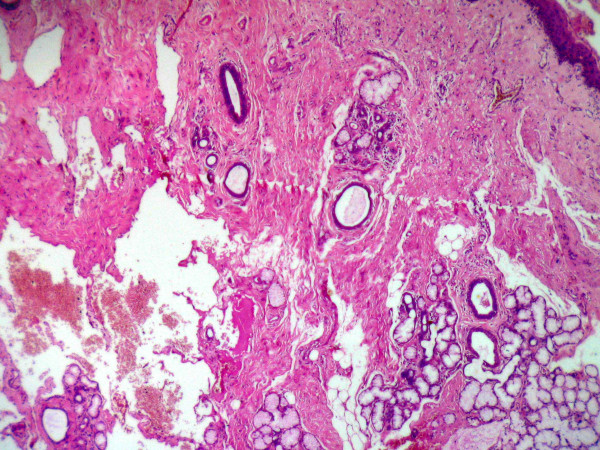
Shows vascular proliferations at a depth from the surface epithelium and displaced residual salivary gland structures (Original maginification × 24).

Routine microscopy showed predominantly entrapped ducts and effaced acini amid proliferating thin walled endothelium lined cavernous type of blood vessels (Fig 1 & Fig 2A,B,C &2D). In other fields, displacement of mucous salivary acini and ducts towards the surface epithelium was evident. The intervening connective tissue between the surface epithelium and the main body of the lesion lacked lesional tissue but showed survival of ducts and dense collagenization. (Fig 3). After ascertaining that the lesion was not associated with any syndrome, it was coded as hemanigoma involving minor salivary glands.

## Discussion

As in other sites, capillary and cavernous are the two main types of hemangiomas that can occur in salivary glands [2]. Cavernous hemangioma is rare but can occur in adults [3]. Capillary hemangioma is more common in childhood but spontaneously regress before adolescence [1-4].

The diagnosis of hemangioma is often challenging when it involves salivary glands [2]. Computed tomography (CT) and magnetic resonance imaging (MRI) may aid in the pre-operative diagnosis of hemangioma but is not entirely reliable to differentiate it from other hypervascular lesions [5]. CT and MRI was not performed in the present case. However, suspicion of a vascular lesion was high based on the clinical findings. Although there was no thrill or bruit, the lesion blanched and refilled on digital pressure. Since conventional radiograph fail to establish intra bony pathology, the lesion was excised. Prolonged ooze (15 minutes) was observed but easily controlled on pressure pack application. At no point of time hemangioma in the vicinity of a minor salivary gland was suspected. This is not surprising given the fact that clinical examination may not easily distinguish salivary tissue from extrasalivary tissue origin [6].

The microscopic features of salivary gland hemangioma is distinctive, and is characterized by effaced acinar structures, retained ducts, and lesional tissue within the confines of salivary gland lobules [1]. Although these histologic features are regarded as diagnostic of primary hemangioma in major salivary glands, it cannot be said of minor salivary glands. This is in part because of lack of encapsulation in minor salivary glands, and thus assessment of origin within the salivary gland connective tissue stroma is rendered more difficult.

In the present case, the presence of vascular proliferations in the main substance of the salivary gland associated with retained ducts and effaced acinar units may indicate intraglandular origin, as it is at a deeper location than one would expect. But in the absence of any specific histological parameters to assess minor salivary gland origin with certainty, it is virtually impossible to exclude secondary involvement [7].

The comparison of the present case with arteriovenous hemangioma merits attention because of the presence of pain, which is a prominent symptom in arteriovenous hemangioma [1] and is possibly related to the presence of neuropeptides within the lesion [8]. However, unlike hemangioma they lack effaced acinar units and retained ducts within the main lesion [1]. Moreover, medium to large sized arteries and veins are an integral part of arteriovenous hemangioma [1,4,8]. Another important discriminator is the presence of intralesional nerve bundles in arteriovenous hemangioma as opposed to the occasional presence of nerve twigs in hemangioma [8]. Neither large diameter vessels nor nerve bundles were identified in the present case. Therefore, this lesion is unlikely to be of arteriovenous hemangioma.

**In conclusion**, the present lesion shows definite involvement of minor salivary gland by a hemangioma, a rare histological finding, which may or may not have originated in the gland.
